# Augmentation versus No Augmentation for Immediate Postextraction Implants

**DOI:** 10.1155/2018/5209108

**Published:** 2018-10-16

**Authors:** Huda Hamed Basher Mohamed, Asma M. Serag Eldien, Amr Zahran

**Affiliations:** ^1^PhD Degree Candidate, Department of Oral Medicine, Oral Diagnosis and Periodontology, Faculty of Oral and Dental Medicine, Cairo University, Giza, Egypt; ^2^Assistant Lecturer, Department of Periodontology, Benghazi University, Benghazi, Libya; ^3^Lecturer, Department of Periodontology, 6 October University, Giza, Egypt; ^4^Professor of Oral Medicine and Periodontology, Faculty of Oral and Dental Medicine, Oral Medicine and Periodontology Department, Cairo University, Giza, Egypt

## Abstract

**Purpose:**

To assess the effects of augmentation versus no augmentation in patients restored with immediate postextraction single-tooth implants on implant failure and patient satisfaction.

**Materials and methods:**

We searched the Cochrane Oral Health Group Trial Register, Cochrane Central Register of Controlled Trials, MEDLINE, and the WHO International Clinical Trial Registry Platform (22 March 2017). Two reviewers independently assessed trials for inclusion and risk of bias, extracted data, and checked for accuracy. We have expressed results as risk ratio or mean differences, together with their 95% confidence intervals.

**Results:**

We included six studies (287 participants). Two trials compared no augmentation versus bone graft augmentation and reported no implant failures in both groups after a follow-up period of 6 months (20 implants) and 1 year (34 implants). One trial compared bone graft augmentation versus membrane augmentation and reported no difference in implant failure between both groups after 6 months (risk ratio (RR) 0.98, 95% confidence interval (CI) 0.06 to 15.31) or 1 year of follow-up (RR 0.33, 95% CI 0.01 to 7.86), and no implants were lost after 3 years. Three trials compared membrane augmentation versus combined bone graft and membrane augmentation, and there was no difference between the groups after six months of follow-up in implant failure (RR 5.13, 95% CI 0.63 to 41.93) or after 1 year (RR 0.38, 95% CI 0.02 to 9.05). There was insufficient evidence regarding patient satisfaction in all the included trials.

**Conclusions:**

In patients restored with immediate postextraction single-tooth implants, there is insufficient evidence to recommend simultaneous augmentation or a certain augmentation protocol to enhance implant survival and patient satisfaction. This trial is registered with PROSPERO (CRD42017054439).

## 1. Background

Immediate implant is defined as the placement of an implant into the fresh socket directly after tooth extraction. This reduces the time from tooth extraction to complete rehabilitation when compared to classical delayed implantation protocols, eliminates the need for the surgical reopening, and reduces the total cost of the treatment [1–4]. In addition, some reports suggest that this procedure helps in preserving the alveolar bone at the extraction sites [5, 6]. However, controversy was raised during the last decade about the fact that immediate implantation can preserve alveolar bone dimension following tooth extraction, and some authors stated that, although immediate implant placement is considered a predictable treatment modality, it does not preserve the alveolar ridge dimension and that bone resorption takes place at the buccal and lingual plates of bone [7, 8].

The discrepancy in size and form between the extraction socket and the implant creates bony defects around the coronal portion of the implant [9]. These defects might affect the long-term success of the implants by interfering with the process of osseointegration and compromise the final esthetic outcome due to the soft tissue recession that follows the resorption of the buccal plate of bone [8, 10].

A variety of regenerative techniques using bone grafts and/or barrier membranes are used to promote bone regeneration in localized defects around implants placed into fresh extraction sockets. The different types of bone graft materials commonly used include autogenous bone grafts, allografts, xenografts, and alloplasts.

All grafting materials regenerate bone through one or more of these three mechanisms: osteogenesis, osteoinduction, and osteoconduction, and how the graft acts is determined by its origin and composition. In osteogenesis, new bone is formed even in the absence of local undifferentiated mesenchymal stem cells, while in osteoinduction, undifferentiated mesenchymal stem cells transform into osteoblasts or chondroblasts through growth factors that exist only in the living bone. In osteoconduction, a bioinert scaffold, or physical matrix, is provided for the deposition of new bone from the surrounding bone or to encourage differentiated mesenchymal cells to grow along the graft surface [11]. Additionally, the presence of membranes around the implants act as a mechanical barrier to prevent soft tissue ingrowth into the bony defect and allow osteogenic cells to populate and seal the gap and develop new bone matrices for osseous regeneration [12, 13].

The use of these regenerative materials was proposed when the size of the residual bone defect exceeded a 1 to 2 mm threshold of horizontal gap between the implant surface and the buccal bony wall. Several authors recommended the use of bone graft in such cases [14–16]. However, the validity of this “dimension” has never been conclusively demonstrated, leading other studies to suggest that such bone defects could heal clinically without any bone regeneration procedures or grafting materials [8, 17]. Nonetheless, these regenerative procedures reduced but did not eliminate the buccal bone resorption [18]. Consequently, there is no current optimal bone augmentation technique regarding graft selection during immediate implant placement [19].

While immediately placed implants are currently gaining wide popularity, there is no agreement on whether these implants need to be augmented or on, the ideal augmenting protocol to be followed. Therefore, a systematic review is needed to assess the effects of different augmentation approaches versus no augmentation on the long-term survival of the implants and the patient satisfaction with this treatment modality.

The aim of this work was to assess the effects of augmentation versus no augmentation in patients restored with immediate postextraction single-tooth implants on implant failure and patient satisfaction.

## 2. Methods and Materials

In this systematic review, two review authors independently assessed study eligibility criteria.

### 2.1. Inclusion Criteria

1. Only randomized controlled trials are included
2. The patients were restored with immediate postextraction single-tooth implant in healthy fresh extraction socket (both arches, all sites, and all implant-loading protocols were included).
3. Human studies

We compared specifically between the following groups:

1. No augmentation versus bone graft augmentation
2. No augmentation versus membrane augmentation
3. No augmentation versus combined bone graft and membrane augmentation
4. Bone graft augmentation versus membrane augmentation
5. Bone graft augmentation versus combined bone graft and membrane augmentation
6. Membrane augmentation versus combined bone graft and membrane augmentation

### 2.2. Exclusion Criteria

1. Unclear information about patient, implant, follow-up, and study design
2. Study in animals
3. Case series/reports
4. Other implant protocols (delayed placement)
5. Postextraction immediate implant in the infected socket.

The primary outcomes were implant failure (total implant loss or nonfunctioning implant) and patient satisfaction. The secondary outcomes included infection, soft tissue recession, and marginal bone loss.

*Time frame*. All the outcomes were assessed at the following time intervals, starting from the time of implant placement.

1. Short term, 1–6 months
2. Medium term, 6–12 months
3. Long term, 1–3 years

### 2.3. Search Strategy

We searched the Cochrane Oral Health Group Trial Register (22 March 2017). The Cochrane Oral Health Group's Trials Register contains trials identified from monthly searches of the Cochrane Central Register of Controlled Trials (CENTRAL), weekly searches of MEDLINE, weekly searches of Embase, and hand searches of journals and the proceedings of major conferences. In addition, we searched CENTRAL (*The Cochrane Library*, 2017, Issue 03), MEDLINE (January 1966 to 22 March 2017), and the WHO International Clinical Trial Registry Platform (22 March 2017) using the search strategies detailed in Appendix. We also hand searched citation lists of relevant publications and included studies. We did not apply any language or date restrictions.

### 2.4. Data Collection

We followed the methods of Rabe et al. [20] and the standard methodological procedures expected by Cochrane (MECIR 2016) [21].

Two authors assessed for inclusion of all potential studies we identified as a result of the search strategy. We resolved any disagreement regarding the selection of the studies through discussion or, if required, we consulted a third person.

We designed a form to extract data. For eligible studies, two review authors extracted the data using the agreed form. There were no discrepancies. We entered data into Review Manager Software [22] and checked for accuracy. When information regarding any of the above was unclear, we contacted the authors of the original reports to provide further details.

### 2.5. Risk of Bias Assessment

Two review authors independently assessed risk of bias for each study using the criteria outlined in the *Cochrane Handbook for Systematic Reviews of Interventions* [23]. There were no disagreements on the assessment of risk of bias in the included studies.

We made explicit judgements about whether studies are at “Low risk,” “High risk,” or “Unclear risk” of bias, according to the criteria given in the handbook [23], regarding the following domains: random sequence generation (checking for possible selection bias), allocation concealment (checking for possible selection bias), blinding of participants and personnel (checking for possible performance bias), blinding of outcome assessment (checking for possible detection bias), incomplete outcome data (checking for possible attrition bias due to the amount, nature, and handling of incomplete outcome data), selective reporting (checking for reporting bias), and other bias (checking for bias due to problems not covered by the previously mentioned domains). With reference to these criteria, we assessed the likely magnitude and direction of the bias and whether we considered it likely to impact the findings. We planned to explore the impact of the level of bias through undertaking sensitivity analyses.

### 2.6. Data Analysis

We carried out statistical analysis using the Review Manager software [22]. For dichotomous data, we presented results as a summary risk ratio with 95% CI, and for continuous data, we used the mean difference with 95% CI. The statistical unit was the patient and not the implants in all the outcomes except “implant failure”, where we considered the number of implants in each group. In trials that compared more than two intervention groups, we combined all the groups with different bone graft materials into one single-“bone graft augmentation” group and all the groups with different membrane materials into one single-“membrane augmentation” group and then made multiple pairwise comparisons between all possible pairs of intervention groups. For included studies, we noted levels of attrition. We contacted the authors for missing data. For all outcomes, we carried out analyses, as far as possible, on an intention-to-treat basis, i.e., we included all participants randomised to each group in the analyses and all participants analysed in the group to which they were allocated, regardless of whether or not they received the allocated intervention. The denominator for each outcome in each trial was calculated as the number randomised minus any participants whose outcomes are known to be missing. We have assessed statistical heterogeneity in each meta-analysis using the Tau-squared (T^2^), I-squared (*I*^2^), and chi-squared (Chi^2^) statistics. We regarded heterogeneity as substantial if *I*^2^ was greater than 30% and either T^2^ was greater than zero, or there was a low *P* value (less than 0.10) in the Chi^2^ test for heterogeneity.

### 2.7. Data Synthesis

We carried out statistical analysis using the Review Manager software [22]. We used a fixed-effect meta-analysis for combining data where it is reasonable to assume that studies were estimating the same underlying treatment effect, i.e., where trials were examining the same intervention, and we judged the trials' populations and methods sufficiently similar. If there was clinical heterogeneity sufficient to expect that the underlying treatment effects differed between trials, or if we detected substantial statistical heterogeneity, we explored this by sensitivity analysis followed by random effects if required. We did not conduct the planned subgroup analyses by the type of loading due to insufficiency of the data.

In future updates of this review, if there are 10 or more studies in the meta-analysis, we will investigate reporting biases (such as publication bias) using funnel plots. We will assess funnel plot asymmetry visually. If asymmetry is suggested by a visual assessment, we will perform exploratory analyses to investigate it.

## 3. Results

**Figure 1:**
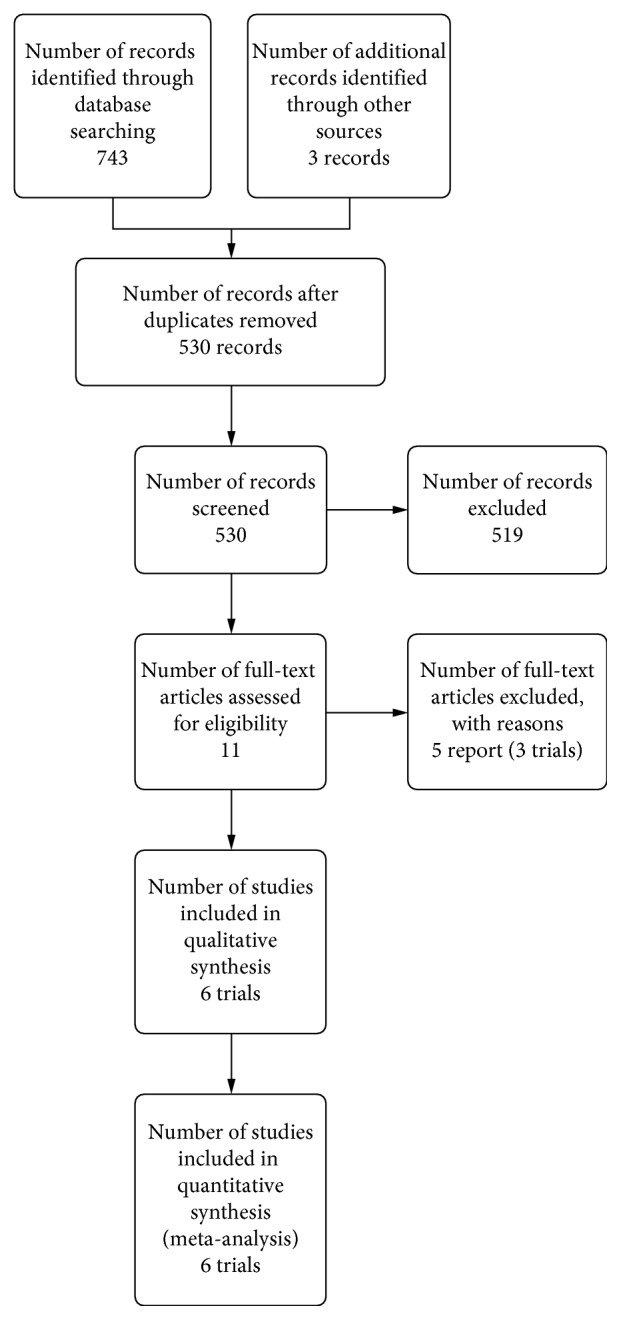
PRISMA flow diagram.

We identified 9 potentially eligible studies (11 reports) [5, 6, 12, 18, 24–30]. The detailed search results are depicted in the PRISMA flow diagram (Figure 1). We included six studies (287 participants) [5, 6, 12, 24–26]. All included patients were restored with immediate postextraction single-tooth implants and randomised to augmentation versus no augmentation. Detailed description of the included studies is shown in Table 1. Three trials were excluded from the review [18, 27, 30] because the studies included implants placed in sites with persistent infection (infected sockets).

### 3.1. Risk of Bias in Included Studies

Three of the included studies described adequate methods of sequence generation [5, 6, 24], while the remaining three studies provided insufficient information on how the sequence was generated and the risk of bias was unclear [12, 25, 26]. Regarding allocation concealment, only one study was at low risk of selection bias [6], while all the other five trials provided no information on how the sequence was concealed [5, 12, 24–26].

Neither the participants nor the caregivers were blinded in the included trials. Given the nature of the intervention, blinding was not feasible, and we considered the risk of performance bias to be low. Regarding detection bias, we assessed blinding separately for different classes of outcomes. We judged the risk of detection bias to be low in objective outcomes and high in patient-reported outcomes since lack of blinding can potentially introduce bias for this class of outcomes through multiple pathways (different expectations from the two groups and biased assessment of the effect) [23].

**Figure 2:**
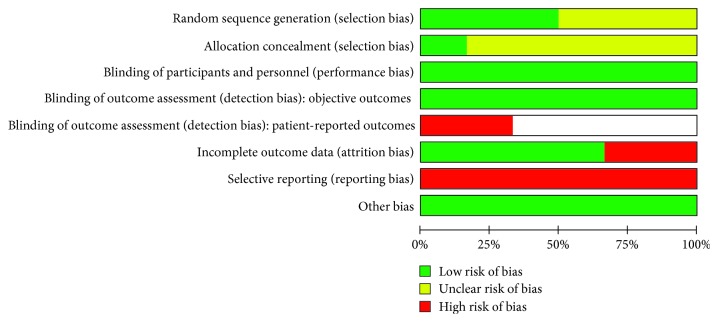
Risk of bias graph: review of authors' judgements about each risk of bias item presented as percentages across all included studies.

**Figure 3:**
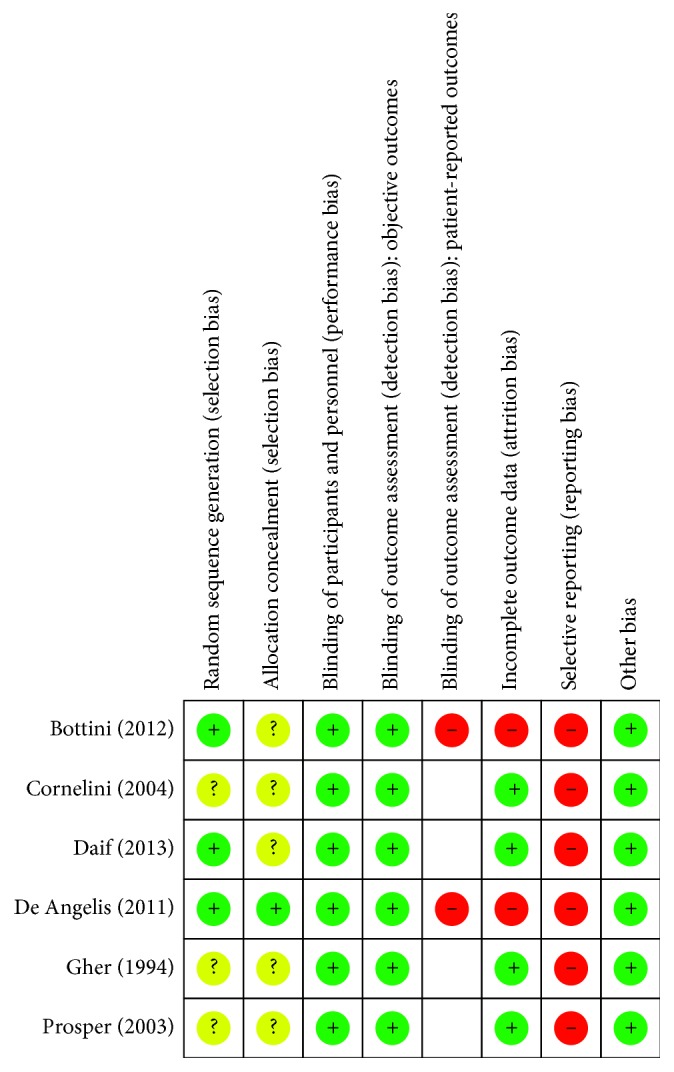
Risk of bias summary: review of authors' judgements about each risk of bias item for each included study.

Two studies were assessed as high risk of attrition bias [6, 24] due to performing per-protocol analysis. The remaining four trials included all randomised participants and were at low risk of attrition bias [5, 14, 21, 26]. All included trials were at high risk of reporting bias. Bottini et al. [24]; Cornelini et al. [25]; Daif [5]; and Gher et al. [12] failed to include results for key outcomes expected to have been reported for such studies, while De Angelis et al. and Prosper et al. [6, 26] reported the outcomes of interest in the review incompletely hindering their use in the analysis. A summary of “Risk of bias” assessments is given in Figures 2 and 3.

### 3.2. Primary Outcomes

#### 3.2.1. Implant Failure

**Figure 4:**
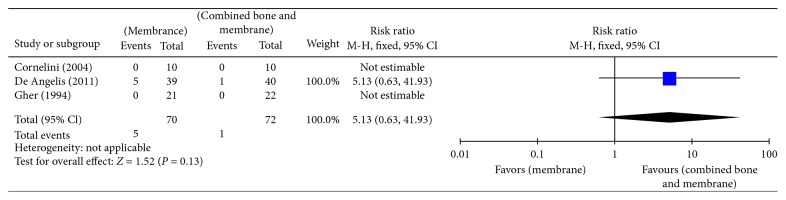
Analysis 1.1: implant failure (short term; 1–6 months).

**Figure 5:**
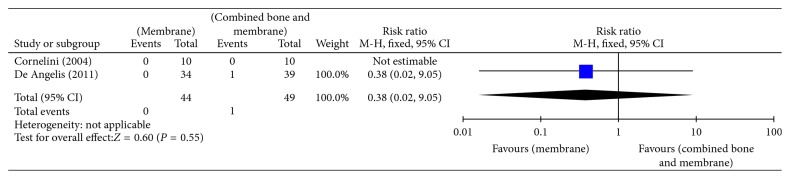
Analysis 1.2: implant failure (medium term; 6–12 months).

Bottini et al. [24] and Daif [5] compared no augmentation with bone graft augmentation and reported no implant failures in both groups after a follow-up period of 6 months (20 implants) and 1 year (34 implants). Prosper et al. [26] compared bone graft augmentation with membrane augmentation and reported no difference in implant failures between both groups after 6 months (RR 0.98, 95% CI 0.06 to 15.31) or 1 year of follow-up (RR 0.33, 95% CI 0.01 to 7.86) (Table 2). No implant failures were reported at 3 years of follow-up. Cornelini et al. [25], De Angelis et al. [6], and Gher et al. [12] compared membrane augmentation with combined bone graft and membrane augmentation and reported no difference in implant failures between both groups after 6 months (RR 5.13, 95% CI 0.63 to 41.93) (Figure 4) or 1 year of follow-up (RR 0.38, 95% CI 0.02 to 9.05) (Figure 5, Analysis 1.2).

#### 3.2.2. Patient Satisfaction

Patient satisfaction was only reported in [6], but no usable data were provided by the trial.

### 3.3. Secondary Outcomes

#### 3.3.1. Infection

**Figure 6:**
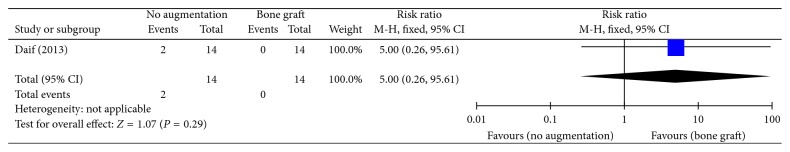
Analysis 1.3: infection (short term; 1–6 months).

**Figure 7:**
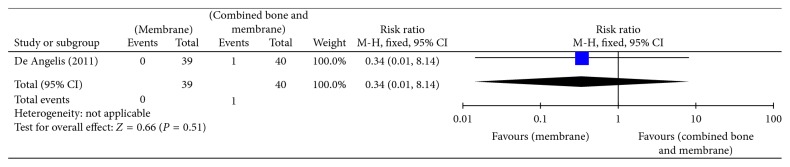
Analysis 1.4. infection (short term; 1–6 months).

Bottini et al. and Daif [5, 24] compared no augmentation with bone graft augmentation, and there was no difference between both groups in infection after 6 months (RR 5.00, 95% CI 0.26 to 95.61) (Figure 6, Analysis 1.3). No cases of infection were reported after 1 year of follow-up. Membrane augmentation and combined bone graft and membrane augmentation were compared by N. De Angelis et al. [6], and there was no difference in infection between both groups after 6 months of follow-up (RR 0.34, 95% CI 0.01 to 8.14) (Figure 7, Analysis 1.4).

#### 3.3.2. Soft-Tissue Recession

**Figure 8:**
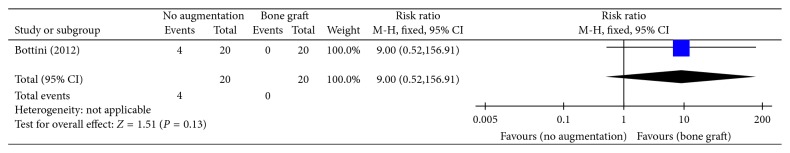
Analysis 1.5. gingival recession (short term; 1–6 months).

This outcome was only reported by Bottini et al. [24] who compared no augmentation with bone graft augmentation. After 6 months of follow-up, there was no difference between both groups in soft-tissue recession (RR 9.00, 95% CI 0.52 to 156.91) (Figure 8).

#### 3.3.3. Marginal Bone Loss

**Figure 9:**
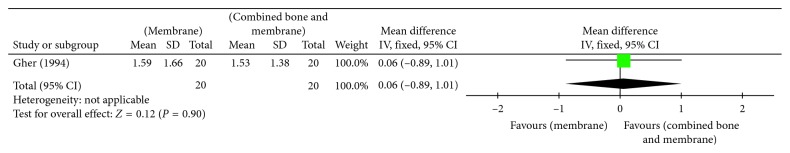
Analysis 1.6. marginal bone loss (short term; 1–6 months).

Only one trial provided usable data regarding marginal bone loss [12]. The trial compared membrane augmentation with combined bone graft and membrane augmentation and reported no difference between both groups after 6 months of follow-up (mean difference (MD) 0.06, 95% CI −0.89 to 1.01) (Figure 9, Analysis 1.6).

## 4. Discussion

A total of six studies (287 participants) reported the effectiveness of augmentation versus no augmentation in patients restored with immediate postextraction single-tooth implants. The included trials compared no augmentation versus bone graft augmentation, bone graft augmentation versus membrane augmentation, and membrane augmentation versus combined bone graft and membrane augmentation. There was no difference in implant failure, infection, soft-tissue recession, or marginal bone loss, and there is insufficient evidence regarding patient satisfaction. No trials compared no augmentation with membrane augmentation, no augmentation with combined bone graft and membrane augmentation, or bone graft augmentation with combined bone graft and membrane augmentation.

The six identified trials were not sufficient to address the review objectives. The trials failed to include most of the outcomes of interest in the review and assessed surrogate measures, and the outcomes investigated were poorly reported. In addition, the number of patients in the individual primary studies was relatively small, which increases the risk of random error. Currently, there is no agreement on whether the immediately placed implants should be augmented or not, or what is the ideal augmenting protocol to be followed.

The results of this review do not allow a robust conclusion regarding the effects of augmentation in patients restored with immediate postextraction single-tooth implants on implant failure and patient satisfaction. Since all the included trials had small sample sizes, and there were few events and the CI included appreciable benefit and harm in implant failure, infection, soft-tissue recession, and marginal bone loss, we would rate down quality of evidence by two levels for imprecision. Additionally, the included trials suffer from serious methodological limitations that are likely to result in a biased assessment of the intervention effect. In most of the studies, it is unclear how randomization (both sequence generation and allocation concealment) was performed, and the risk of reporting bias was high. Accordingly, the overall quality of evidence across reported outcomes was downgraded to very low quality of evidence according to the GRADE approach for grading evidence (we have very little confidence in the effect estimate: the true effect is likely to be substantially different from the estimate of effect) [31].

We were able to identify all relevant studies and obtain all relevant data. We did not apply date or language restrictions to our search. Two review authors assessed eligibility for inclusion, carried out data extraction, and assessed risk of bias. Accordingly, we are not concerned that the methods used in the review could have introduced bias.

Three previous systematic reviews have addressed the question of the effectiveness of augmentation around immediately placed implants and the preferred augmenting protocol [32–34], one of which is a Cochrane systematic review [30]. It was in agreement with our results and concluded that it is unclear whether augmentation procedures are needed with immediate single implants placed in fresh extraction sockets. Chen and Buser [32] assessed the influence of simultaneous bone augmentation procedures on the esthetic outcomes of implants placed in postextraction sites, and Lin et al. [34] investigated the effect of various surgical and restorative interventions on the midbuccal mucosal level in immediately placed implants. Both reviews did not find sufficient evidence on the effects of augmentation or the augmenting protocol employed on the soft-tissue recession or the esthetic outcome of the treatment.

## 5. Conclusions

In patients restored with immediate postextraction single-tooth implants, there is insufficient evidence to recommend simultaneous augmentation or a certain augmentation protocol to enhance implant survival and patient satisfaction. More well-designed and well-conducted randomised controlled trials (RCTs), with appropriate a priori calculated sample sizes, are needed to determine whether immediately placed implants need to be augmented and to answer the question of the ideal augmenting protocol to be followed. The trials should assess long-term patient relevant outcomes and properly report these outcomes to allow their inclusion into future analysis.

## Figures and Tables

**Table 1:** Characteristics of included studies.

Study ID	Methods	Participants	Mean age (range), years	Interventions	Outcomes	Follow-up	Note
Bottini, 2012	Parallel design RCT where patients were randomly assigned to the two treatment groups	40 (17F and 23M)	22–80, mean (45–65)	Group 1: immediate implants were inserted in association with a deantigenated collagenated bone substitute of porcine origin and were maintained in place by the use of equine collagen sponge. Group 2: immediate implants were placed with no grafting material	The measurement of the buccolingual width, implant mobility, pain, suppuration, and peri-implant radiolucency	6-month	Italy

Cornelini, 2004	Parallel design RCT where patients were randomly assigned to treatment groups	20 (11F-9M)	21–60, mean 45	Group 1: immediate implants with Bio-Oss (bovine-derived porous bone mineral matrix) covered by Bio-Gide membrane (pure collagen membrane). Group 2: immediate implants with Bio-Gide membrane alone.	Proximal radiographic bone level, mucosal coverage of the implant, probing attachment level, oral hygiene status (plaque score) and soft tissue condition (mucositis score)	6-month	Country not stated

Daif, 2013	Parallel design RCT where patients were randomly divided into two equal groups	28 (18F and 10M)	22–48, mean 34	Group 1: immediate implants with pure-phase multiporous beta-TCP particles. Group 2: immediate implants with no filling materials	Bone density, implant failure, and infection	3 and 6 months after loading (implant loading three months after surgery)	Egypt

De Angelis, 2011	Multicenter parallel group RCT. Four computer-generated restricted random lists were created	80	—	Group 1: immediate implant with the bone substitute granules (Endobon®; Biomet 3i) and a resorbable collagen barrier (OsseoGuard®, Biomet 3i). Group 2: immediate implants with a resorbable collagen barrier (OsseoGuard®, Biomet 3i) alone	Implant failures, any biological or biomechanical complications, peri-implant marginal bone levels, esthetic evaluation of the vestibular and occlusal clinical pictures, and patient satisfaction	1 year after loading (implant loading after 3-4 months)	Italy

Gher, 1994	Factorial design RCT. The patients were randomly assigned to four treatment groups according to implant type and use or nonuse of DFDBA at the time of implant placement	36	—	Group 1: Titanium plasma-sprayed implant 3 without DFDBA. Group 2: Titanium plasma-sprayed implant with DFDBA. Group 3: Hydroxyapatite-coated implant 1 without DFDBA. Group 4: Hydroxyapatite-coated implant with DFDBA	Implant failure and crestal bone apposition measurements	6-month	USA

Prosper, 2003	Parallel design RCT. The patients were divided randomly into 2 groups	83	21–75	Group 1: immediate implants in combination with the use of synthetic hydroxyapatite and patients. Group 2: immediate implants combined with a bioabsorbable membrane based on polyglycolic and polylactic acid copolymers	Soft-tissue examination, implant mobility, bone loss, and implant success	Every 3 months for an overall period of 4 years	Italy

**Table 2:** Bone graft augmentation versus membrane augmentation.

Outcome	Studies	Participants	Statistical method	Effect estimate
Implant failure (short term; 1–6 months)	Prosper et al. [26]	111	Risk ratio (M-H, fixed, 95% CI)	0.98 [0.06, 15.31]
Implant failure (medium term; 6–12 months)	Prosper et al. [26]	109	Risk ratio (M-H, fixed, 95% CI)	0.33 [0.01, 7.86]

## Appendix

Search strategy: (randomized controlled trial [Publication Type] OR controlled clinical trial [Publication Type] OR randomized [Title/Abstract] OR randomly [Title/Abstract] OR trial[Title/Abstract] OR groups[Title/Abstract] NOT animals [MeSH Terms] NOT humans [MeSH Terms]AND Dental Implants [MeSH Terms] OR Dental Implants [Title/Abstract] OR AND Bone Transplantation [MeSH Terms] OR Bone Transplantation [Title/Abstract] OR Bone Grafting [Title/Abstract] OR Guided Tissue Regeneration [MeSH Terms] OR Guided Tissue Regeneration [Title/Abstract] OR Membrane Grafting [Title/Abstract] OR Membrane Augmentation [Title/Abstract]).
